# Moyamoya Disease and Steno-Occlusive Disease in a Young Stroke Patient: A Case Report

**DOI:** 10.7759/cureus.37909

**Published:** 2023-04-20

**Authors:** Heng-Tien Aaron Lee, Hazem Abosheaishaa, Mahmoud Nassar, Merjona Saliaj

**Affiliations:** 1 Internal Medicine, St. George's University School of Medicine, True Blue, GRD; 2 Internal Medicine, Icahn School of Medicine at Mount Sinai, Queens Hospital Center, New York, USA; 3 Internal Medicine, Gastroenterology, Cairo University, Cairo, EGY; 4 Internal Medicine, Icahn School of Medicine at Mount Sinai, New York City (NYC) Health+Hospitals-Queens, New York, USA; 5 Medicine, Icahn School of Medicine at Mount Sinai, Queens Hospital Center, New York, USA

**Keywords:** global aphasia, hemicraniectomy, intracranial arteries, cerebrovascular disorder, moyamoya disease

## Abstract

Moyamoya disease is a rare cerebrovascular disorder characterized by progressive stenosis and occlusion of the intracranial arteries, resulting in the formation of collateral vessels. We present a case of a 24-year-old South Asian female with no prior medical history who presented with persistent headaches, right-hand numbness and pain, and global aphasia. Imaging revealed severe steno-occlusive disease involving the left internal carotid artery terminus, the proximal middle cerebral artery (MCA), and the anterior cerebral artery. The patient underwent a hemicraniectomy due to malignant MCA syndrome and was prescribed aspirin and fluoxetine. Further evaluation with a cerebral angiogram revealed severe steno-occlusive disease involving the left internal carotid artery terminus, the proximal middle cerebral artery, and the anterior cerebral artery. The patient had Moyamoya disease. This case emphasizes the necessity of including Moyamoya disease in the differential diagnosis, as it can result in serious neurological impairments.

## Introduction

Moyamoya disease is a rare cerebrovascular disorder that affects the intracranial arteries, forming collateral vessels. The disease was first described in Japan by Takeuchi and Shimizu in 1957 [1]. The name "Moyamoya" is derived from the Japanese term for a "puff of smoke," which describes the appearance of the collateral vessels on angiography [2]. Although the exact etiology of the disease remains unknown, genetic and environmental factors have been implicated in its development [3]. Moyamoya disease is most commonly diagnosed in children and young adults and is associated with an increased risk of stroke, cognitive impairment, and other neurological deficits [4]. Early recognition and appropriate treatment are critical in preventing stroke and improving outcomes for patients with this rare condition.

## Case presentation

We present the case of a 24-year-old South Asian female with no known medical history who presented to the emergency department with persistent headaches for three days. Upon arrival, the patient was awake but unresponsive to verbal questions and unable to complete sentences. The patient's husband reported that she had experienced right-hand numbness and pain. The patient's family denied vomiting, seizures, or psychiatric conditions. The patient was also unable to recall her husband's name.

Physical examination

Upon physical examination, the heart rate was 71 beats per minute. Blood pressure was 118/77 mmHg, temperature was 97.9°F, and respiratory rate was 18 breaths per minute. The patient could follow certain commands, including smiling and blowing, but had weak grip strength in her right hand. The patient's pupils responded equally to light and accommodation, and her face was symmetrical. The strength in the left upper extremity was five out of five, and the strength in the right upper extremity was four out of five. A deep tendon reflex of 2+ was recorded in the right lower extremity and a reflex of 1+ in the left lower extremity. The Babinski sign was not observed. The complete blood count, basic metabolic panel, and urine analysis were all within normal ranges. The beta-human chorionic gonadotropin level (beta-HCG) was negative. The erythrocyte sedimentation rate (ESR) was 29.

Diagnostic imaging

**Figure 1:**
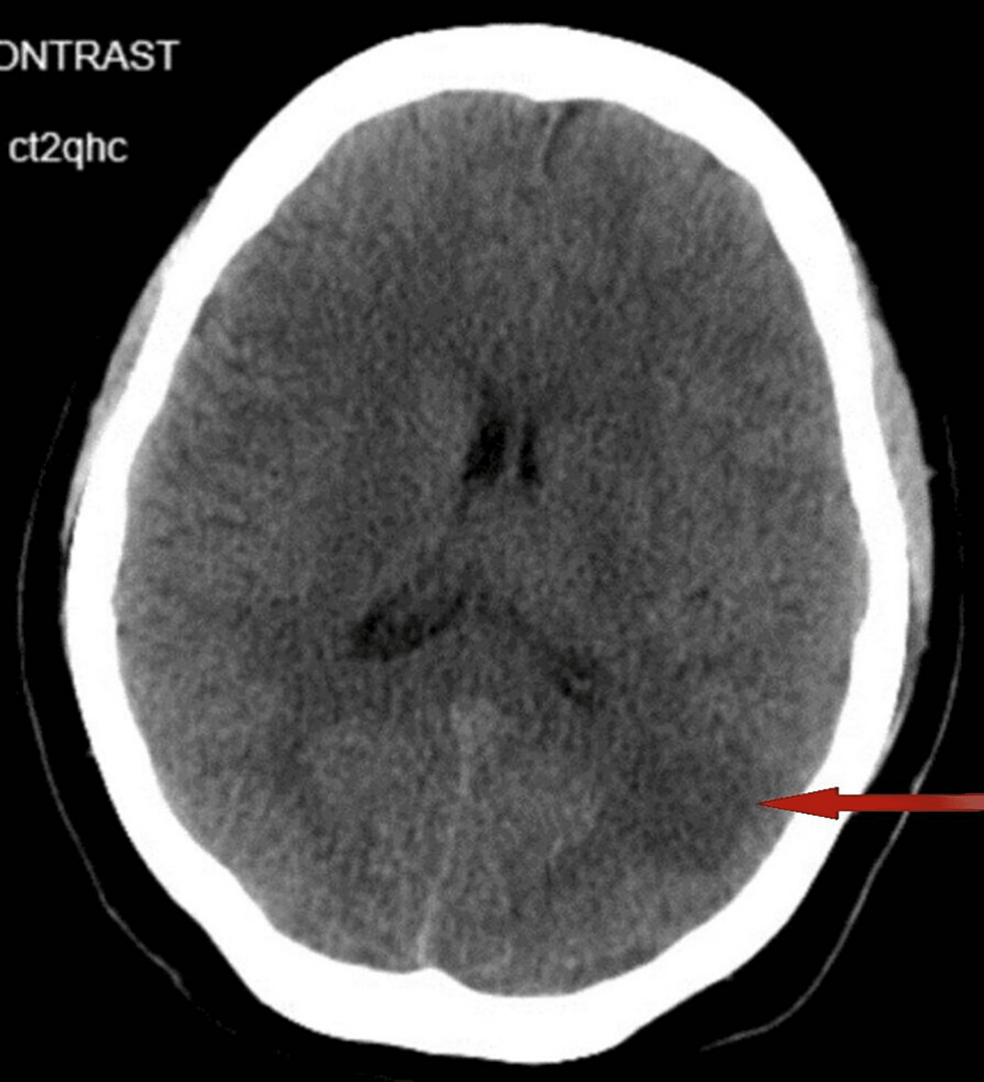
Masslike rounded area of hypoattenuation in the posterior left temporoparietal region.

The radiological report indicates a non-contrast CT scan showed hypoattenuation in the left cerebral hemisphere, suggesting possible neoplasms, metastases, infection foci, or ischemic/infarcted areas (Figure 1). A small 2 mm mass may be present in the interpeduncular space. CT angiogram and magnetic resonance angiography (MRA) imaging revealed attenuation in the left internal carotid artery (ICA) and associated branches, suggesting occlusive or near-occlusive thrombosis and severe stenosis. A large area of restricted water diffusion was observed, consistent with acute infarction in the left middle cerebral artery (MCA) territory. There was also a 2 mm rightward shift in the midline structure. The findings suggest a left middle cerebral artery infarction that is potentially related to Moyamoya disease.

Treatment and follow-up

The patient underwent a hemicraniectomy due to malignant MCA syndrome. Following surgery, the patient exhibited global aphasia but could speak single words such as "home" and "yes." The patient has been prescribed fluoxetine 20 mg for motor recovery and depression. Additionally, the patient is taking aspirin 81 mg, heparin 5000 units per mL, sennosides 8.8 mg/5 mL; syrup 17.6 mg, esomeprazole 40 mg, and ondansetron 4 mg. After that, the patient was admitted to an acute rehabilitation facility. The patient continued to exhibit right flaccid hemiplegia caused by a stroke, along with apraxia, improving aphasia, sustained clonus of the right ankle and wrist, increased tone and spasticity in the right pronators and biceps and triceps, minimal activation of the right hip flexors with gravity eliminated, and the ability to follow some one-step commands. She was advised to continue with aspirin 81 mg, fluoxetine 20 mg, which would be escalated to 40 mg if tolerable, gabapentin 300 mg qHS for sleep and neuropathic pain, and enoxaparin 40 mg a day.

Further evaluation was conducted with a femoral cerebral angiogram, which revealed severe steno-occlusive disease involving the left internal carotid artery terminus, the proximal middle cerebral artery, and the anterior cerebral artery. Most of the flow from the internal carotid artery was directed into the neovascularization networks, resulting in the appearance of a puff of smoke, which is consistent with Moyamoya disease. The patient had no visible anterior cerebral artery A1 on the left, while the visible lenticulostriate medially and the anterior choroidal artery exhibited some hypertrophy. The right inferior cerebral artery circulation, specifically the ipsilateral posterior circulation, was extensively collateralized via pial collaterals. The left anterior cerebral artery is filled through a robust anterior communicating artery. The right A1 anterior cerebral artery was mildly narrowed. This appearance is consistent with left ICA steno-occlusive disease associated with Moyamoya disease.

## Discussion

The Moyamoya disease, initially described in 1957 as "hypoplasia of the bilateral internal carotid arteries," is a cerebrovascular disorder that affects the cerebral arteries, particularly those of the internal carotid artery, leading to steno-occlusive lesions [3]. The condition's name originates from the angiographic appearance of an aberrantly dilated network of collateral vessels, which is said to resemble "something hazy, like cigarette smoke puff," meaning Moyamoya in Japanese [2].

Moyamoya disease presents with a wide range of symptoms, including transient ischemic attacks to permanent neurological deficits caused by either ischemic or hemorrhagic stroke [5]. While initially believed to affect mostly Asians, the disease has now been reported in people of various ethnic backgrounds, including American and European populations [6]. The incidence of the disease is estimated to be 0.5-15% per 100,000 people in Asian populations and 1% in other geographies, such as North America [7].

Due to the increasing number of accidental discoveries of asymptomatic Moyamoya cases and the documented stroke cases diagnosed with Moyamoya disease over the last decade, it has been suggested that the condition may be an underdiagnosed or unidentified cause of stroke [8]. Consequently, patients who exhibit unexplained symptoms of cerebral ischemia should be suspected of having Moyamoya. The diagnosis of Moyamoya can be made using various imaging modalities, including CT, CTA, magnetic resonance imaging (MRI), and MRA; however, a definitive diagnosis is achieved via formal angiography, which reveals a unique arteriographic presentation characterized by distal intracranial internal carotid artery stricture that extends to the proximal anterior and middle arteries [3].

Currently, no medication available can cure the condition or prevent an imminent stroke by improving blood flow to the affected brain areas. However, antiplatelets can be used to prevent an ischemic stroke from microthrombi in stenosed areas [9]. Calcium channel blockers have been used to prevent headaches and migraines in such patients [10]. Revascularization surgery has been found to improve symptoms in a significant number of patients with Moyamoya, with 87% of patients endorsing symptomatic improvement in one study [11].

Many cases of Moyamoya have been reported in patients with Down syndrome, which may be attributed to their increased risk of developing cardiovascular diseases and congenital anomalies of the circle of Willis [12]. Furthermore, many cases have been reported with Graves' disease, which may be explained by the proposed cause of Moyamoya disease, linked to inherited genetic and immunogenic factors. Ten percent of Moyamoya patients in Japan have a positive family history, and T-cell dysregulation has been suggested as a common pathogenic link between cell growth and vascular dysregulation in Moyamoya and immune and inflammatory stimulation of the thyroid in Graves' disease [13].

## Conclusions

Moyamoya disease is an uncommon, progressive cerebrovascular disorder that can cause stroke in children and adults. Although genetic factors are thought to be involved, the disease's underlying cause is still not completely understood. Early detection and prompt treatment are essential for preventing stroke and reducing the possibility of long-term neurological damage. The current standard of care involves revascularization procedures, but ongoing research is looking into alternative therapies like medical management and gene therapy. The general public and healthcare professionals need to be more knowledgeable about Moyamoya disease to treat this potentially fatal condition quickly and effectively.
